# Optical Genome Mapping for the Identification of Complex Structural Variants in Hereditary Angioedema

**DOI:** 10.1007/s10875-026-02015-z

**Published:** 2026-03-28

**Authors:** Laura Batlle-Masó, Kornelia Neveling, Johana Gil-Serrano, Eveline Kamping, Amber den Ouden, Raoul Timmermans, Aina Bruch-Tàrrega, Aina Aguiló-Cucurull, Janire Perurena-Prieto, Paula Fernández-Álvarez, Marloes Steehouwer, Alexander Hoischen, Mar Guilarte, Roger Colobran

**Affiliations:** 1 Translational Immunology Research Group, Vall d’Hebron Research Institute (VHIR), Vall d’Hebron Barcelona Hospital Campus, Barcelona, Catalonia Spain; 2 Pediatric Infectious Diseases and Immunodeficiencies Unit, Vall d’Hebron Children’s Hospital (HUVH), Vall d’Hebron Barcelona Hospital Campus, Barcelona, Catalonia Spain; 3 Jeffrey Modell Diagnostic and Research Center for Primary Immunodeficiencies, Barcelona, Catalonia Spain; 4 Department of Human Genetics, Radboud University Medical Center, Nijmegen, the Netherlands; 5 Allergy Department, National Reference Hereditary Angioedema Center (CSUR), Vall d’Hebron Barcelona Hospital Campus, Barcelona, Catalonia Spain; 6 Allergy Research Unit, Vall d’Hebron Research Institute (VHIR), Vall d’Hebron Barcelona Hospital Campus, Barcelona, Catalonia Spain; 7 Immunology Division, Vall d’Hebron University Hospital (HUVH), Vall d’Hebron Barcelona Hospital Campus, Barcelona, Catalonia Spain; 8 Department of Clinical and Molecular Genetics, Hospital Universitari Vall d’Hebron (HUVH), Vall d’Hebron Barcelona Hospital Campus, Barcelona, Catalonia Spain; 9 Department of Internal Medicine, Radboud Expertise Center for Immunodeficiency and Autoinflammation, Radboud University Medical Center, Radboud Center for Infectious Disease (RCI), Nijmegen, the Netherlands; 10 Department of Cell Biology, Physiology and Immunology, Autonomous University of Barcelona (UAB), Bellaterra, Catalonia Spain

**Keywords:** Hereditary angioedema, C1 inhibitor deficiency, structural variants, optical genome mapping, mobile elements, SINE-VNTR-Alu (SVA) element

## Abstract

**Purpose:**

Hereditary angioedema due to C1-inhibitor deficiency (HAE-C1INH) is caused by heterozygous pathogenic variants in the *SERPING1* gene. Structural variants (SVs) account for 10–15% of cases, and while many can be identified using conventional diagnostic approaches such as multiplex ligation-dependent probe amplification (MLPA), some remain difficult to detect. This study aims to assess the utility of optical genome mapping (OGM) for the detection of both typically described and atypical structural variants in HAE-C1INH.

**Methods:**

MLPA, OGM, long-read whole-genome sequencing (LR-WGS), Sanger sequencing and cDNA analysis.

**Results:**

First, we assessed whether OGM could detect common SVs in HAE-C1INH affecting the *SERPING1* coding region. We tested a family previously diagnosed by MLPA with a large heterozygous deletion encompassing exons 1–2 of *SERPING1*. OGM successfully identified this deletion in both affected members. Next, we analyzed a three-generation HAE-C1INH family with no genetic findings after Sanger sequencing and MLPA. OGM revealed a 3.8 kb insertion in *SERPING1*, which was further characterized using LR-WGS as a SINE-VNTR-Alu (SVA) mobile element inserted in intron 7, demonstrating the complementary role of LR-WGS in precisely defining the nature and location of SVs detected by OGM. Finally, we demonstrated that the allele carrying the SVA insertion was absent in cDNA resulting in *SERPING1* functional haploinsufficiency in this family.

**Conclusion:**

OGM is a reliable and robust technique for detecting SVs affecting both coding and non-coding regions in HAE-C1INH. Using OGM, we report the first case of HAE-C1INH caused by the insertion of an SVA mobile element.

**Supplementary Information:**

The online version contains supplementary material available at 10.1007/s10875-026-02015-z.

## Introduction

Hereditary angioedema (HAE) is a rare disorder affecting approximately 1 in 50,000 individuals worldwide [1]. It is characterized by recurrent episodes of swelling that vary in trigger, frequency, and severity, and may result in life-threatening airway obstruction. Most cases of HAE result from uncontrolled bradykinin production due to heterozygous loss-of-function genetic variants in *SERPING1*, which encodes the C1 inhibitor protein (C1INH). C1INH concentration and function are ordinarily assessed when HAE is suspected, and if C1INH function is reduced -regardless of whether the C1INH concentration is also decreased- the diagnosis of HAE-C1INH is confirmed. In such cases, sequencing of *SERPING1* (by Sanger or NGS), typically reveal pathogenic point mutations or small indels. However, up to 10% of patients may remain genetically undiagnosed due to the presence of structural variants (SVs), which are not detectable by Sanger sequencing and may be missed by conventional short-read NGS [2]. This relatively high proportion of SVs in HAE-C1INH can be attributed to intrinsic characteristics of *SERPING1*, including its genomic location and sequence composition [3–5]. Classically, the gold standard technique for identifying SVs in HAE-C1INH, as well as in other human diseases, has been multiplex ligation-dependent probe amplification (MLPA) [5, 6]. MLPA uses probe pairs that hybridize to adjacent target sites; only correctly ligated probes are amplified and quantified, allowing for simultaneous analysis of multiple targets in a single reaction [7]. Although MLPA is useful for identifying SVs not detectable by standard sequencing methods, it typically detects only copy number variations (CNVs), such as deletions or duplications affecting exonic regions. This represents a limitation, as balanced SVs (e.g., inversions and translocations) and variants in non-coding regions (e.g., introns or the promoter) may remain undetected.

We aimed to demonstrate the utility of a relatively novel technique, optical genome mapping (OGM), for identifying SVs in HAE-C1INH. OGM is a next-generation cytogenomic technology designed to detect SVs in the genome with high resolution and accuracy. Unlike traditional sequencing, which analyzes short DNA fragments, OGM uses ultra-high molecular weight DNA molecules labeled at specific 6-mer sequence motif. These molecules are linearized and imaged as they pass through nanochannels, generating optical maps that represent the physical layout of the genome [8]. OGM can identify a wide range of SVs -including insertions, deletions, duplications, inversions, and translocations- across both coding and non-coding regions [9]. It is particularly valuable for detecting variants that are difficult to identify using conventional short-read sequencing or methods like MLPA. The technique does not require prior knowledge of the target region, allowing for genome-wide analysis in a single experiment.

In the context of HAE-C1INH, OGM offers a promising approach to uncovering cryptic or complex structural alterations in the *SERPING1* gene that may underlie cases unresolved by standard genetic testing. In this study, we evaluated the ability of OGM to detect SVs in HAE-C1INH. First, we demonstrated that OGM can successfully identify CNVs affecting exonic regions, which are the most common SVs in HAE-C1INH. Next, we applied OGM to investigate a three-generation family with HAE-C1INH, who had remained undiagnosed for over 20 years. OGM identified a large insertion in the non-coding region of the *SERPING1* gene. Using long-read whole genome sequencing, we confirmed that this insertion corresponded to a mobile element located in intron 7 of *SERPING1*, leading to functional haploinsufficiency and causing HAE-C1INH. Our findings highlight the utility of OGM in detecting SVs in HAE-C1INH and provide, for the first time, a description of a mobile element insertion as the cause of the disease.

## Methods

### Patients and Samples

This study included five patients from two unrelated families. Family A comprised two affected individuals from two consecutive generations, while Family B included three affected individuals spanning three consecutive generations. All patients were attended at the Allergy Division of Vall d’Hebron University Hospital (Barcelona, Spain). Written informed consent for the studies reported here and for publication of the article was obtained from all patients, according to the procedures of the Clinical Research Ethics Committee of the Vall d’Hebron University Hospital [code: PR(AG)202/2021].

### Laboratory Tests

Serum levels of C4, and C1-INH were determined by turbidimetry (Optilite, Binding Site, UK). C1-INH functional activity was determined by ELISA (QuidelOrtho, USA).

### Multiplex Ligation-Dependent Probe Amplification

Multiplex ligation-dependent probe amplification (MLPA) was employed to identify CNVs affecting the coding region of *SERPING1* using the Salsa MLPA Probemix P243 kit (MRC Holland), following the manufacturer’s protocol. The probe mix includes 11 control probes, 8 probes targeting *SERPING1*, 13 probes for *F12*, and 1 probe for *APLNR*, a gene located upstream of *SERPING1*. Four DNA samples without CNVs in this region were used as controls alongside patient samples. Fragment separation was performed on an ABI 3500 Genetic Analyzer (Applied Biosystems), and data analysis was conducted using Coffalyser software version 220513.1739.

### Optical Genome Mapping

Optical genome mapping (OGM) was performed as described previously [10], with minor modifications. In brief, ultra-high molecular weight DNA (UHMW-DNA) was isolated from peripheral blood (stabilized with EDTA) using the SP-G2 Blood & Cell Culture DNA Isolation Kit (Bionano Genomics, San Diego, CA, USA). Labeling of isolated UHMW-DNA was performed using the Direct label and stain (DLS) kit (Bionano Genomics, San Diego, CA, USA). 750ng Labeled DNA was analyzed on a Saphyr system, using G3.3 chips. *De novo* assembly and variant calling was done with Bionano Solve v3.7 and Bionano Access v1.7.2. Variants were filtered for being present in less than 1% of a Bionano control database (containing approximately 300 samples).

### Long Read Whole Genome Sequencing

Long read whole genome sequencing (LR-WGS) was performed using SPRQ chemistry and a PacBio Revio system (PacBio, Menlo Park, CA, USA). For this, 2.6ug UHMW-DNA (left-over from OGM UHMW isolation) was first pre-sheared by pipetting and vortexing. Library preparation was performed with the Revio SPRQ HiFI prep kit 96 with a Hamilton Star V liquid handler following the protocol “Preparing whole genome libraries using the HiFi prep kit 96, Procedure & checklist ” (103-420-700 REV04 APR2025). Two additional samples were pooled together and sequenced on the a Revio system using the Revio SPRQ Sequencing plate (250 pm loading, 24 hour movie time), leading to an average coverage of ~ 10x for our sample. Data analysis was performed as described by Höps et al. [11]. The region of interest in *SERPING1* was manually checked in the bam files opened in IGV (Integrated Genomics Viewer, v2.17.0). From there, the insertion sequence was copied to Dfam (release 3.9) to investigate the source of inserted sequence.

### *SERPING1* cDNA Analysis

Upon identifying a mobile element insertion in intron 7 of the *SERPING1* gene, we investigated potential alterations in RNA processing. Total blood was collected using Tempus™ Blood RNA Tubes (Thermo Fisher Scientific, USA) and total RNA was isolated using the Tempus™ Spin RNA Isolation Kit (Thermo Fisher Scientific, USA). cDNA was synthesized from 1 µg of RNA using anchored-oligo(dT)18 primers and with the Transcriptor First Strand cDNA Synthesis Kit (Roche, Switzerland). The *SERPING1* cDNA region spanning exons 6 to 8 was amplified by PCR, and the resulting products were purified and sequenced using the Sanger method on an Applied Biosystems 3500 Genetic Analyzer (Thermo Fisher Scientific, USA). Primer sequences and PCR conditions are available upon request.

## Results

### Optical Genome Mapping Can Detect Structural Variants in the *SERPING1* Gene

**Fig. 1:**
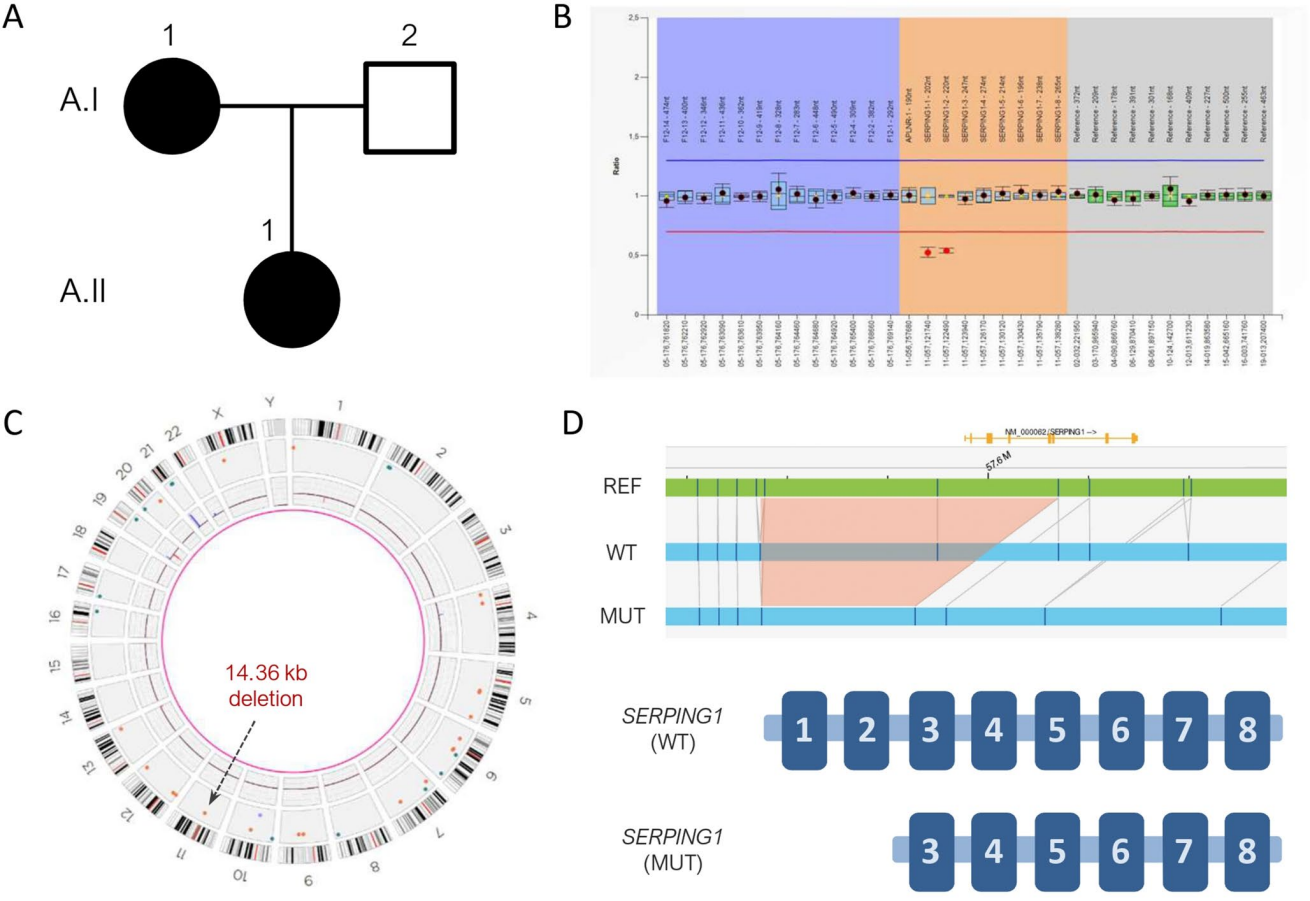
Identification of *SERPING1* coding-region copy number variants by optical genome mapping.** (A)** Pedigree of Family A; black circles indicate affected individuals. **(B)** MLPA results for patient A-II.1 (identical results were obtained for A-I.1). Red circles highlight probes with significantly reduced signal, consistent with a deletion. **(C)** Optical genome mapping results for patient A-II.1 represented as a circos plot. A 14.36 kb deletion encompassing part of the *SERPING1* gene is indicated by an orange dot and marked with an arrow (identical results were obtained for A-I.1). **(D)** Screenshot of optical genome mapping results visualized using Bionano Access software. The reference genome is shown in green; patient alleles are shown in blue. A schematic representation of the wild-type (WT) and mutated (MUT) *SERPING1* alleles in the patients is included

While OGM has proven to be a powerful method for detecting SVs, it may have reduced sensitivity in genomic regions near centromeres [10]. The *SERPING1* gene is located at 11q12.1, relatively close to the centromere of chromosome 11, particularly when compared to most genes on the long (q) arm. Given this proximity, our initial objective was to determine whether OGM can reliably detect SVs affecting *SERPING1*. From our cohort of patients with HAE, we selected a family (Family A) in whom a structural variant affecting the *SERPING1* gene had recently been identified in our centre. Family A includes two individuals diagnosed with HAE-C1INH: a 59-year-old mother (A-I.1) and her daughter (A-II.1) (Fig. 1A; Table 1). The mother (A-I.1) presented disease onset at 20 years of age, with recurrent swelling episodes predominantly affecting peripheral and abdominal regions, lasting around 72 h, with no identified triggers and no relevant comorbidities. The daughter (A-II.1), currently 23 years old, experienced her first angioedema episodes at 4 years of age, involving peripheral, abdominal, and genital regions, with episodes lasting approximately 96 h; stress was reported as a common trigger, and no relevant medical history was noted. In this family, Sanger sequencing yielded negative results. Subsequently, MLPA identified a heterozygous deletion encompassing the first two exons of *SERPING1* (Fig. 1B). This large deletion was classified as pathogenic and considered causative of HAE-C1INH in this family. We performed OGM on the two affected members of Family A. Analysis of the OGM data revealed a 14.36 kb deletion on chromosome 11 that was absent from public databases (Fig. 1C). Closer inspection confirmed that the deletion encompassed the 5′ upstream regulatory region and part of the coding sequence of *SERPING1* (Fig. 1D). These findings demonstrate that OGM is capable of detecting CNVs affecting the coding region of *SERPING1* gene.

**Table 1:** Overview of laboratory and molecular analyses performed in the patients.

**Patient**	**C4**(mg/dL)(ref: 10-40)	**C1-INH **(mg/dL)(ref: 15-35)	**C1-INH**(UC1-In/mL)(ref: 0.7-1.3)	**Sanger ** ***SERPING1***	**MLPA ** ***SERPING1***	**OGM**	**LR-WGS**
A-I.1	3.1 ↓	7.9 ↓	0.2 ↓	Negative	Del exon 1-2	14.36 kb deletion	-
A-II.1	7.5	6.1 ↓	0.4 ↓	Negative	Del exon 1-2	14.36 kb deletion	-
B-I.1		5.1 ↓	0.1 ↓	Negative	Negative	-	-
B-II.2		6.2 ↓	0.4 ↓	Negative	-	-	-
B-III.1		6.9 ↓	0.6 ↓	Negative	Negative	3.8 kb insertion	3.6kb insertion

### OGM in Genetically Unresolved HAE-C1INH: Detection of a Non-coding *SERPING1* Structural Variant

**Fig. 2:**
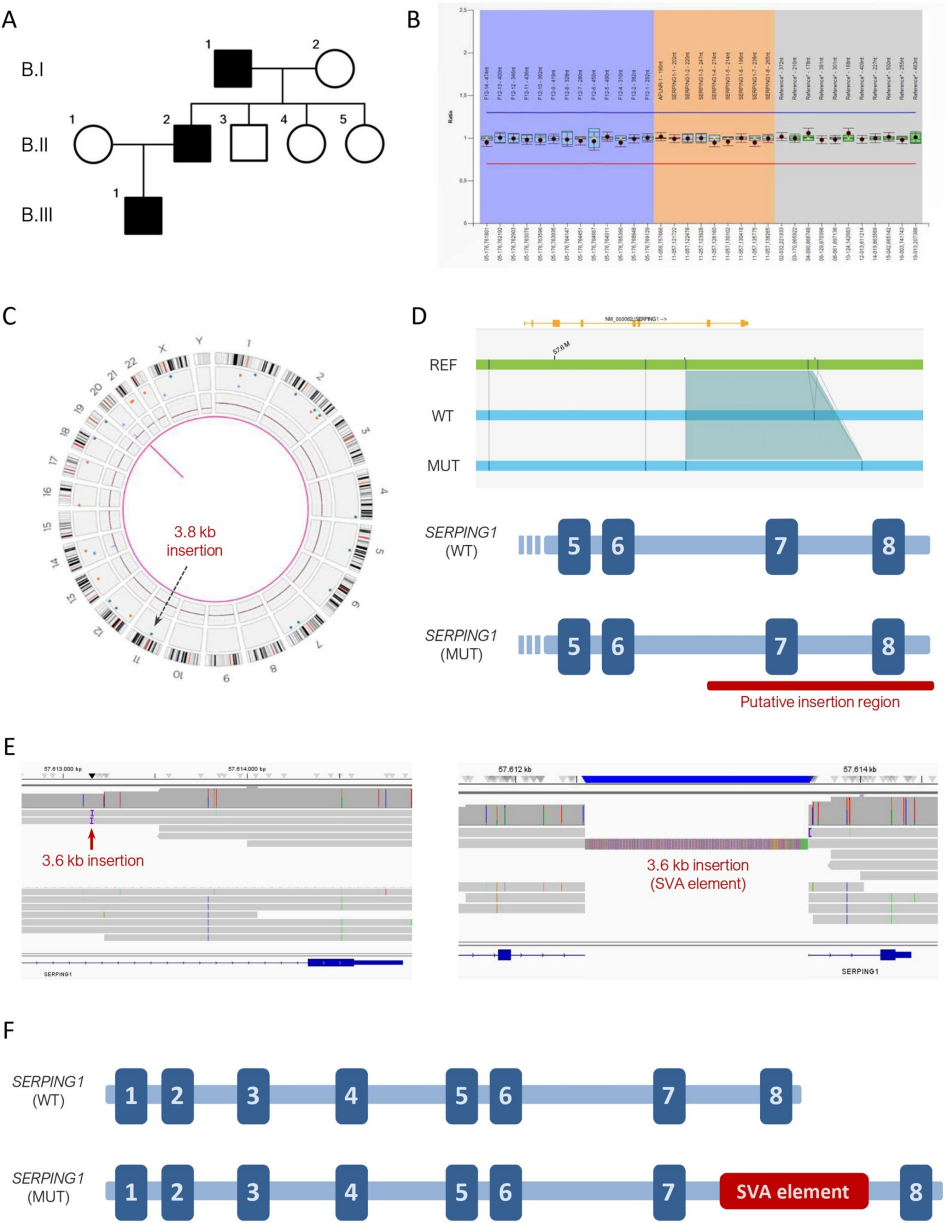
Identification and characterization of a mobile element insertion in the non-coding region of *SERPING1*. **(A)** Pedigree of Family B; black squares indicate affected individuals. **(B)** MLPA results showing no copy number alterations in patient B-II.2. **(C)** Optical genome mapping results for patient B-II.2 represented as a circos plot. A 3.8 kb insertion involving the *SERPING1* gene is indicated by a green dot and marked with an arrow. **(D)** Screenshot of optical genome mapping results visualized using Bionano Access software. The reference genome is shown in green; patient alleles are shown in blue. A schematic representation of the wild-type (WT) and mutated (MUT) *SERPING1* alleles is included. In the mutated allele, the region where the insertion is potentially located is highlighted with a red bar. **(E)** Screenshot of long-read whole-genome sequencing results visualized in the Integrative Genomics Viewer (IGV). Two out of eight reads covering *SERPING1* intron 7 display a 3.6 kb insertion (left). The inserted region, corresponding to an SVA element, is shown in the expanded view (right). **(F)** Schematic representation of the WT and MUT *SERPING1* alleles in patient B-II.2. The SVA element insertion within intron 7 is depicted in the MUT allele

To explore the potential of OGM in patients without a prior genetic diagnosis, we selected a family (Family B) with HAE-C1INH affecting three consecutive generations (Fig. 2A). This family has been followed at our hospital for over 20 years without a confirmed genetic diagnosis. The first patient (B-I.1), an 83-year-old male with a history of hypertension and atrial fibrillation, experienced disease onset at 16 years of age, with recurrent peripheral and abdominal angioedema episodes, without identified triggers. The second patient (B-II.2), a 55-year-old male with no relevant comorbidities, presented disease onset at 20 years, with predominantly abdominal, genital, and peripheral attacks; stress and trauma were identified as common triggers. The third patient (B-III.1), a 21-year-old male with a background of asthma and allergic rhinitis, experienced onset at 10 years of age, with peripheral and abdominal attacks lasting approximately 72 h; stress, trauma, and infections were reported as common triggers. Sanger sequencing and MLPA were performed in multiple family members, but no pathogenic variants were identified (Fig. 2B; Table 1). We performed OGM in patient B-II.2 and identified a heterozygous insertion of approximately 3.8 kb on chromosome 11 that was absent from public databases (Fig. 2C). The insertion was mapped to a region between intron 6 and the 3′ downstream sequence of *SERPING1* (Fig. 2D), although the exact breakpoint could not be precisely determined due to the intrinsic limitations of the OGM technique. Since this variant had not been detected by MLPA, we hypothesized that it was located within intronic regions (intron 6 or intron 7) or, less likely, in the 3′ downstream region. Although the insertion putatively lies in a non-coding region, its size and position suggest that it could disrupt *SERPING1* transcription and/or splicing. We therefore considered this insertion to be the likely cause of HAE-C1INH in this family.

### Identification of an RNA Retrotransposon Insertion in *SERPING1* by Long-read Whole Genome Sequencing

To determine the exact location and sequence of the insertion detected by OGM, we performed long-read whole-genome sequencing (LR-WGS) on patient B-II.2. We obtained approximately 10x coverage in the target region, with two reads supporting the insertion. The insertion was located in intron 7 and measured 3.6 kb in length (Fig. 2E). Sequence analysis revealed that the inserted fragment corresponded to a mobile element, specifically a SINE-VNTR-Alu (SVA) element (Fig. 2E and Figure S1). SVA elements are RNA retrotransposons, meaning they can copy and insert themselves into new genomic locations via an RNA intermediate. These elements are human-specific and relatively young in evolutionary terms, making them significant contributors to genomic variation and, in some cases, human disease [12]. In this family, an SVA element was inserted into intron 7 of one of the *SERPING1* alleles (Fig. 2F), presumably causing HAE-C1INH.

### SVA Insertion in *SERPING1* Intron 7 Causes Functional Haploinsufficiency

**Fig. 3:**
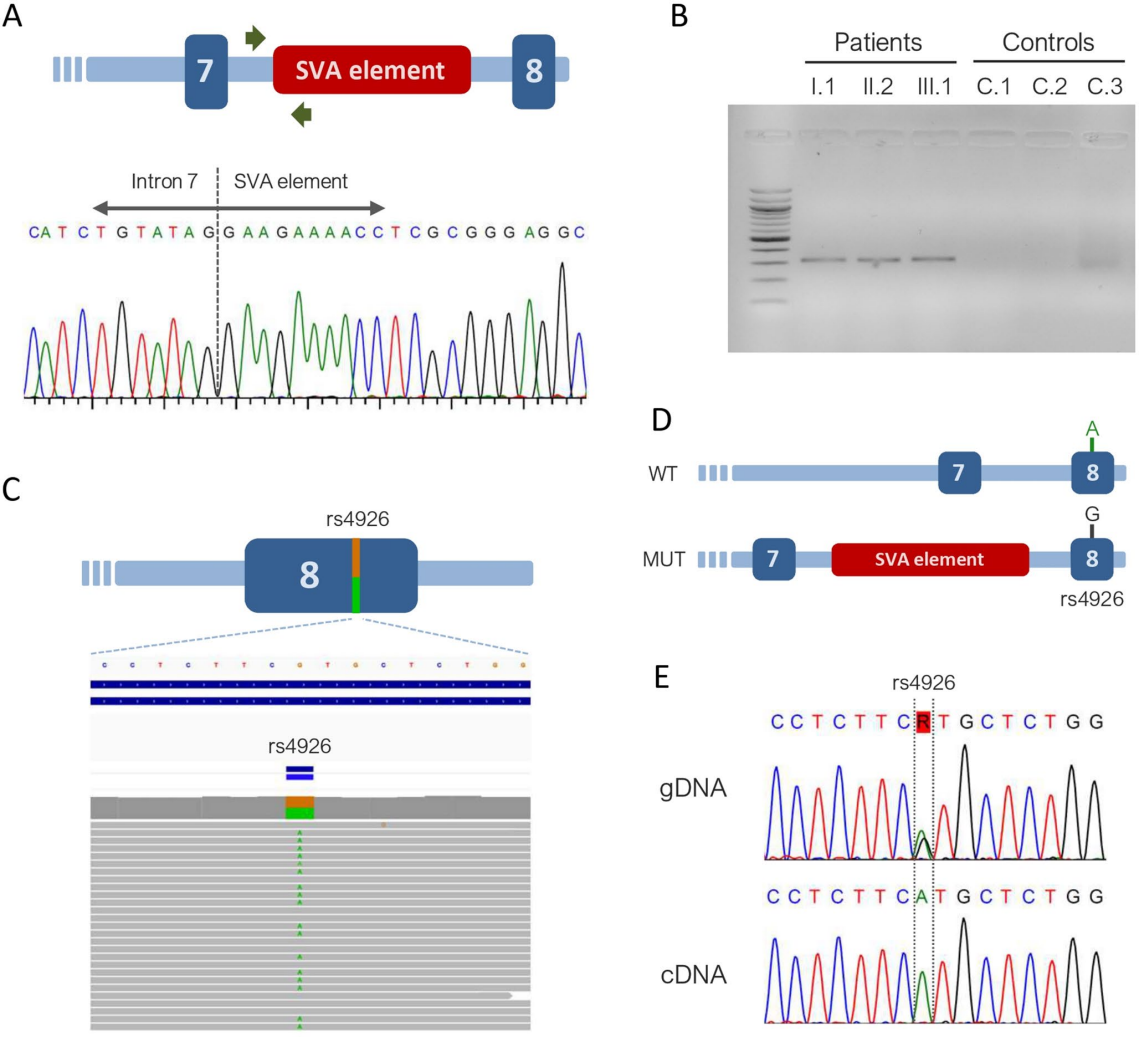
SVA insertion in *SERPING1* intron 7 causes functional haploinsufficiency in Family B.** (A)** Design of a specific PCR assay to detect the SVA element. The locations of the primers are indicated by green arrows. Sanger sequencing shows the 5′ breakpoint of the SVA insertion in *SERPING1* intron 7 in patient B-II.2. **(B)** Agarose gel electrophoresis of PCR amplicons from the three affected members of Family B and three healthy controls. Only affected individuals display the expected band, indicating the presence of the SVA insertion in *SERPING1* intron 7. **(C)** Schematic representation of *SERPING1* exon 8 showing that patient B-II.2 is heterozygous for the rs4926 SNP. The reference allele (G) is highlighted in brown, and the alternative allele (A) in green. **(D)** Representation of the wild-type (WT) and mutated (MUT) *SERPING1* alleles in patient B-II.2. Long-read whole-genome sequencing allowed phasing of the rs4926 G allele to the allele carrying the SVA insertion. **(E)** Sanger sequencing of genomic DNA (gDNA) and complementary DNA (cDNA) from patient B-II.2. While both rs4926 alleles (G and A) are present in gDNA, the G allele -corresponding to the allele with the SVA insertion- is absent in cDNA, resulting in functional haploinsufficiency

Since OGM and LR-WGS were only performed in patient B-II.2, we next aimed to confirm that all affected members of Family B carried the SVA insertion. To this end, we designed a PCR assay targeting the 5′ breakpoint of the SVA element, with the forward primer located in intron 7 of *SERPING1* and the reverse primer located within the 5′ region of the SVA sequence (Fig. 3A). All affected family members tested positive for the expected PCR product, confirming the presence of the SVA insertion in *SERPING1* intron 7. In contrast, no amplification was observed in any of the control samples (*n* = 3) (Fig. 3B).

Finally, we aimed to assess the functional impact of the SVA insertion on the affected *SERPING1* allele. Since the SVA was inserted in intron 7, we hypothesized that it could affect *SERPING1* splicing. To test this, we extracted cDNA from total blood samples of patients B-II.2 and B-III.1 and amplified a fragment of *SERPING1* cDNA spanning exons 6 to 8. Both patients, as well as two control samples, produced the expected PCR product, with no additional bands observed in the patient samples (data not shown). Sanger sequencing of the PCR products revealed no alterations, with a single, unaltered sequence from exon 6 to exon 8 (data not shown). Thus, the amplification and sequencing of *SERPING1* cDNA did not reveal any splicing defects. Most splicing defects lead to aberrant transcripts containing premature stop codons, which are typically degraded via nonsense-mediated mRNA decay (NMD). Given that *SERPING1* is highly sensitive to NMD [13, 14], we hypothesized that any aberrant transcripts resulting from the allele with the SVA insertion may be completely degraded, meaning we were only analyzing the wild-type allele in the PCR and sequencing cDNA-based experiments.

To test this hypothesis, we first searched for heterozygous single-nucleotide polymorphisms (SNPs) in the coding region of *SERPING1* in the three affected family members. We found that patient B-II.2 was heterozygous for the rs4926 SNP (Fig. 3C). This is a common population variant (minor allele frequency = 0.27 in Europeans, according to gnomAD v4.1.0), located in exon 8 (c.1438G > A), and results in a missense change (p.Val480Met). No other heterozygous coding SNPs were identified in the remaining patients. Using the LR-WGS data, we established that the G allele of rs4926 was present on the *SERPING1* allele carrying the SVA insertion, while the A allele was on the wild-type allele (Fig. 3D). Next, we sequenced exon 8 of *SERPING1* using both gDNA and cDNA from patient B-II.2. In gDNA, both alleles (G/A) were equally represented, whereas in cDNA, only the A allele –corresponding to the wild-type allele– was detected (Fig. 3E). These results indicate that the transcript from the allele carrying the SVA insertion was absent, likely due to aberrant splicing and degradation via NMD. Taken together, these findings demonstrate that the SVA insertion in intron 7 of *SERPING1* causes functional haploinsufficiency, leading to HAE-C1INH in this family. We classified this variant as pathogenic and submitted to ClinVar (ID:4686556).

## Discussion

In this study, we report for the first time the application of OGM for the detection of SVs in patients with HAE-C1INH. Although OGM has previously been shown to be effective in identifying SVs in other human genetic disorders [15–17], we considered it particularly important to demonstrate its utility in the context of HAE-C1INH. Approximately 10% of patients with HAE-C1INH harbor large genomic rearrangements in the *SERPING1* gene. This phenomenon can be attributed to the genomic architecture of *SERPING1*, which is enriched in repetitive elements such as Alu sequences [5]. These repeats predispose the region to non-allelic homologous recombination and other mechanisms of genomic instability, leading to deletions, duplications, and other structural variants. Among these SVs, CNVs affecting the coding region (i.e., exons) represent a significant subset of pathogenic alterations in *SERPING1* and are routinely detected using MLPA. However, MLPA is limited to detecting unbalanced variants and may fail to identify balanced or non-coding SVs. In this context, OGM offers added diagnostic value by enabling the detection of a broader range of SVs, including those missed by conventional techniques.

We first demonstrated that OGM can detect exonic CNVs in a family in which a heterozygous deletion involving the first two exons of *SERPING1* had previously been identified by MLPA. This proof-of-concept was particularly relevant given that one known limitation of OGM is its reduced sensitivity in genomic regions located near centromeres [10]. These pericentromeric regions are typically rich in highly repetitive sequences, such as satellite DNA, which complicate accurate mapping and alignment. As a result, SVs occurring in or near these regions may go undetected, and this limitation should be considered when interpreting negative OGM results. *SERPING1* is located approximately 3 megabases distal to the centromere on the long arm (q arm) of chromosome 11. Although this places it relatively close to the centromere, it lies outside the pericentromeric region and is generally accessible to most genomic analysis methods, including OGM, albeit with potential sensitivity constraints. Our results in Family A demonstrate that OGM is capable of detecting common exonic CNVs in *SERPING1*, supporting its utility in the genetic diagnosis of structural variants in HAE-C1INH.

We then demonstrated that OGM can resolve previously unsolved HAE-C1INH cases involving uncommon SVs. In Family B, we identified an insertion in *SERPING1* intron 7, which was ultimately characterized as an SVA element. To our knowledge, this is the first report of a mobile element insertion as a disease-causing mechanism in HAE-C1INH. Mobile elements, also known as transposable elements, are DNA sequences capable of changing their position within the genome. They make up nearly half of the human genome and are classified into two major groups: retrotransposons (which move via an RNA intermediate) and DNA transposons (which move directly as DNA, although they are largely inactive in humans) [18]. Among retrotransposons, the most prominent families are long interspersed nuclear elements (LINEs), short interspersed nuclear elements (SINEs; e.g., Alu elements), and SVA elements. While most mobile elements are no longer active, a small number (e.g. SVAs) retain the ability to mobilize. Insertion of these elements into new genomic locations can disrupt gene function or regulation, thereby contributing to human disease [18–20]. SVAs are evolutionarily young, hominid-specific and considered non-autonomous retrotransposons, meaning they cannot mobilize on their own. Instead, they rely on the enzymatic machinery provided by LINE-1 elements [12]. Although SVAs (~ 2,700 copies per human genome) are far less abundant than LINE-1 (> 500,000 copies) or Alu elements (> 1,000,000 copies), they can still cause human disease as their insertion can disrupt coding sequences, alter splicing, or interfere with gene regulation [12, 18]. Pathogenic SVA insertions have been reported in a range of disorders, including neurodevelopmental and neuromuscular diseases [21, 22], retinal conditions [23], and predisposition to cancer [24]. Their complex structure and repetitive nature make them difficult to detect with conventional sequencing approaches, likely leading to underdiagnosis. Emerging technologies such as OGM are therefore particularly valuable for identifying SVA-mediated pathogenic variants. In the family reported here, the SVA element caused HAE-C1INH via insertional mutagenesis, a process in which a mobile element inserts into or near a coding or regulatory region, thereby disrupting gene expression or splicing. Since the SVA element was inserted into intron 7 of *SERPING1*, we hypothesized that the disease-causing mechanism involved disruption of RNA splicing. The insertion of a large DNA fragment − 3.6 kb in length– into an intron only 2.4 kb long could interfere with normal splicing through several mechanisms, including the introduction of cryptic splice sites, disruption of splicing regulatory elements, or alteration of RNA secondary structure. Although we could not identify the exact splicing defect caused by the SVA insertion, cDNA analysis revealed a complete absence of correctly spliced RNA from the allele harboring the SVA. This supports a model of functional *SERPING1* haploinsufficiency as the underlying cause of HAE-C1INH in this family.

Despite the high resolution and long-range capabilities of OGM, several limitations must be acknowledged:

1. Limited detection of small variants: OGM cannot identify single nucleotide variants (SNVs) or small insertions and deletions (indels), as it lacks base-pair resolution. Its resolution is primarily determined by the density of sequence-specific labeling motifs (typically a 6-base pair motif) used during DNA labeling. Regions lacking these motifs may be poorly represented or unmapped. The lower size limit for the reliable detection of insertions and deletions by OGM is approximately 500 bp. Fortunately, the vast majority of SVs identified in patients with HAE-C1INH are larger than 500 bp and are therefore detectable by OGM [25].
2. Limited breakpoint resolution: The precise localization of structural variant breakpoints in OGM depends on the presence and density of label motifs near the breakpoint sites. In the two families reported here, OGM successfully identified a structural variant but was unable to accurately resolve the exact breakpoint positions. This limitation is also encounter when using MLPA.
3. Lack of sequence information: Unlike sequencing, OGM (and MLPA) do not provide nucleotide-level sequence data, as it detects structural changes based on physical maps rather than DNA base reads. While this limitation is often not critical, it can hinder variant characterization in certain cases. For example, in Family B of our study, this limitation initially precluded the identification of the nature of an insertion.

However, many of these challenges can be mitigated by integrating OGM with complementary technologies. For instance, combining OGM with LR-WGS offers both nucleotide-level resolution and long-range genomic context, enabling a more comprehensive and accurate characterization of SVs. In Family B, this combined approach allowed us to precisely resolve the breakpoints and determine the sequence of the insertion.

A limitation of this study is the inclusion of only two families. This small sample size primarily reflects the rarity of HAE-C1INH; furthermore, structural variants account for only 8–10% of the genetic diagnoses in this condition. Despite this limitation, the findings from the two families reported here strongly support the robustness of OGM as a molecular diagnostic tool for HAE-C1INH. Nevertheless, larger validation studies will be necessary to further confirm and generalize these results.

In summary, we demonstrated that OGM is a reliable technology for the identification of both common and complex SVs in HAE-C1INH. This has important implication in diagnosis, since SVs are a quite common type of genetic alteration in HAE-C1INH. Although MLPA, commonly used in genetic diagnosis of HAE-C1INH, is a valuable technique for the detection of CNVs affecting coding regions, it has inherent limitations, particularly in resolving complex or atypical structural variants and alterations outside the targeted probe regions. In contrast, OGM enables unbiased, genome-wide detection of structural variants, including CNVs, with high resolution and without prior knowledge of the affected region. This broader detection capacity allows OGM to overcome some of the constraints of MLPA, potentially leading to the identification of pathogenic variants in patients who remain undiagnosed with conventional methods. As such, OGM can serve as a complementary approach to enhance the diagnostic yield in hereditary angioedema and help ensure that no patients with HAE-C1INH remain without a genetic diagnosis.

## Supplementary Information

Below is the link to the electronic supplementary material.

Supplementary Material 1

## Data Availability

Data is provided within the manuscript or supplementary information files.
