# Supplementary material for: Optical Genome Mapping for the Identification of Complex Structural Variants in Hereditary Angioedema
Source: J Clin Immunol. 2026 Mar 28;46(1):46. doi: 10.1007/s10875-026-02015-z (PMC13149560; doi:10.1007/s10875-026-02015-z)
Supplement: Supplementary file 1 — Supplementary Material 1 [file 10875_2026_2015_MOESM1_ESM.pdf]

Figure S1. Insertion of the SVA element in intron 7 of *SERPING1*.

The full sequence of *SERPING1* intron 7 is shown in black. Canonical donor and acceptor splice sites are highlighted in bold. The inserted SVA element sequence is displayed in blue at the precise integration site.

GTGAGCTCTGGCAGCTTAGGGTTACTCCCAGGCCATCAGAGGAGAAAGGGGGGATCCCTAAGAT
GTAGTTAGCATTCTCTAGAGTATTTTTTACATCCATAATCTCAGTTTGTCCTGCAACCCTGCAA
GTTAGAGGGGTAGGTGCTATTATCCCATTGGATCATTGTGGAAATGGAGGCTCAGAAGCTTTAT
GTGACTTACCCAGAGATTCCATGATTTACCCTTGAGCTAGTCAGAGGCAAACCAGAACACCGAC
CCAGGTCTCCAGCTCTCCTGAGTTTTTTCTATGGTTCCTTGTGACATAGGCAGTGGAACAGTGG
GACAGGTAACCGAGGTGAATTATGGATGCTCCTTCCCCAGACACATTTCAAAACAGTCAGCCAC
CCCGTGGAATATGCAAGAAGCTATCTGGAAGTGCAGATCTGGAGGCTTCTGAGTTTACGAGAGG
CAACAGAGACTCCATTTTCTTTTTTTTTTTTTTTTTTGAGACGGAGTCTTGCTCTGTCACCCAG
GACAGGCTGGAGTGCAGTGGTGCGATCTCTGCTCACTGCAAGATCCGCCTCCCAGATTCACGCC
ATGCCATTCTCTCGCCTCAGCCTCCCGAGTAGCTGGGACTACAGGTGCCCGCCACCACGCCTGG
CTAATTTTGTTTTTGTATTTTTAGTAGAGACGGGGTTTCACTGTGTTAACCAGGATGGTCACAA
TCTCCGGACCTTGTGATCCACCTGCCTCGGCCTCCCAAAGTGCTGGGATTACAGGCATGAGTCA
CCGCGCCCAGCCCAGAGACTCCATTTTCTAACCTTTGTTTTTTTGTTTGTTTGTTTTTGAGACG
GAGCCTCACTCTGTCACCCAGGCTGGAGCGCAGTGGCATGATCTTGGGTCACTGCAACCTCCGC
CTCCTGGGTTCAAGTGATTCTCCTACCTCAGCCTCCCAAGTAGCTGGGATTACAGGCACCTACC
ACCACGCCCAGCTAATTTTTTTGTATTTTTAGTAGAGATGGGGTTTCACCATGTTGGTGAAGCT
GGTCTTGAACTCCTGGGCTCAAGTGATCCACCCACCTCGGCTTCCCAAAGTGCTGGGATTACAG
GTGTGAGCCACTGTGCCCAGCCTCATTTTCTAACCTTTGATCTCATGTCCAGCCCTGTCACTTC
ATTTCTTGTTAGAGAATTTGACCGCCTAACACATGATTCCATTTCCTTGTATATGTCATCTGTA
TAGAAGAAAACCTCGCGGGAGGCAGCGGCTGGAGGAGCGGACGGGCCCCGCGGGGCCCGAGGGC
AAGGAGCAGCCGCCTGCCTTGGCCTCCCAAAGTGCCGAGATTGCAGCCTCTGCCCGGCCGCCAC
CCCGTCTGGGAAGTGAGGAGTGTCTCTGCCTGGCCGTCCATCGTCTGGGATGTGAGGAGCCCCT
CTGCCTGGCTGCCCAGTCTGGAAAGTGAGGAGCGTCTCCGCCCGGCCGCCATCCCATCTAGGAA
GTGAGGAGCGCCTCTTCCCAGCCGCCATCACATCTAGGAAGTGAGGAGCGTCTCTGCCCGGCCG
CCCATCGTCTGAGATGTGGGGAGCGGCCTCTGCCCCGCCGCCCCATCTGGGATGTGAGGAGCGC
CTCTGCCCGGCCGAGACCCCGTCTGGGAGGTGAGGAGCGTCTCTGCCCGGCCGCCCCGTCTGAG
AAGTGAGGAGACCCTCTGCCTAGCAACCACCCCGTCTGAGAAGTGAGGAGTCCCTCCGCCCGGC
AGCCGCCCCGTCTGAGAAGTGAGGAGCCTCTCCACCCGGCAGCCACCCCATCTGGGAAGTGAGG
AGCGTCTCCGCCCGGCAGCCACCCCGTCCGGGAGGGAGGTGGGGGGGGGGTCAGCCCTCCCGCC
CGGCCAGCCGCCCCGTCCGGGAGGGAGGTGGGGGGTCAGTCCCCCGCCTGGCCAGCGTGCCGT
CCAGGAGGGAGGTGGGGGGTCAGCCCCCCGCCCGGCCAGCCGCCCCGTCCGGGAGGTGAGGGG
CGCCTCTGCCCGGCCGCCCCTACTGGGAAGTGAGGAGCCCCTCAGCCCGGCCAGCCACCCCGTC
CGGGAGGGAGATGGGGGGTCAGCCCCCCACCCGGCCAGCCACCCCGTCCGGGAGGGAGGTGG
GGGGGTCAGCCCCCCGCCTGGCCAGCCGCCCCGTCCGGGAGGGAGGTGGGGGGTCAGCCCTCC
GCCCGGCCAGCCGCCCCGTCTGGGAGGTGAGGGGCGCCTCTGCCCGGCCGCCCCTACTGGGAAG
TGAGGAGCCCCTCTGCCCGGCCAGCCGCCCCGTCCGGGAGGGAGGTGGGGGGTCAGCCCCCCG
CCCGGCCAGCCGCCCTGTCCGGGAGGGAGGTGGGGGGTCAGCCCTCCGCTGGGCCAGCCGCCC
CGTCTGGGAGGTGAGGGGCGCCTCTGCCCGGCCGCCCCTACTGGGAAGTGAGGAGCCCCTCTGC
CCGGCCAGCCGCCCCGTCCGGGAGGGAGGTGGGGGGTCAGCCCCCCGCCCGGCCAGCCGCCCT
GTCCGGGAGGGAGGTGGGGGGTCAGCCCTCCGCCGGGCCAGCCGCCCCGTCTGGGAGGTGAG
GGGCGCCTCTGCCCGGCCGCCCCTACTGGGAAGTGAGGAGCCCCTCTGCCCGGCCAGCCGCCCC
GTCCGGGAGGGAGGTGGGGGGTCGGCCCCCCGCCCGGCCAGCCGCCCCGTCCGGGAGGGAGGT
GGGGGGGTCGGCCCCCCGCCCGGCCAGCCGCCCCGTCCGGGAGGGAGGTGGGGGGTCGGCCCC
CCGCCCGGCCAGCCGCCCCGTCCGGGAGGGAGGTGTGGGGGGGTCGGCCCCCCTGCCCGGCCAG
CCGCCCCGTCCGGGAGGTGAGGGGCGCCTCTGCCCGGCCGCCCCTACTGGGAAGTGAGGAGCCC
CTCTGCCCGGCCAGCCGCCCCGTCCGGGAGGGAGGTGGGGGCGTCAGCCCCCCGCCCGGCCAGC

CGCCCCGTCCAGGAGGGAGGTGGGGGGGGGGGTCAGCCCCCCCGCCCGGCCAGCCGCCCCATCC
GGGAGGGAGGTGGGGGGGTCAGTCCCCCGCCTGGCCAGCCGTGCCGTCCGGGAGGGAGGTGGGG
GGGTCAGCCCCCCGCCCGGCCAGCCGCCACGTCCGGGAGGTGAGGGGCGCCTCTGCCCGGCCGC
CCCTACTGGGAAGTGAGGAGCCCCTCAGCCCGGCCAGCCACCCCGTCCGGGAGGGAGATGGGGG
GGTCAGCCCCCCCACCCGGCCAGCCACCCCGTCCGGGAGGGAGGTGGGGGGGTCAGCCCCCCGC
CTGGCCAGCCGCCCCGTCCGGGAGGGAGGTGGGGGGGTCAGCCCTCCGCCCCGGCCAGCCGCCC
CGTCTGGGAGGTGAGGGGCGCCTCTGCCCGGCCGCCCCTACTGGGAAGTGAGGAGCCCCTCTGC
CCGGCCAGCCGCCCCGTCCGGGAGGGAGGTGGGGGGGTCAGCCCCCCGCCCGGCCAGCCGCCCT
GTCCGGGAGGGAGGTGGGGGGTCAGCCCTCCGCTGGGCCAGCCGCCCCGTCTGGGAGGTGAGGG
GCGCCTCTGCCCGGCCGCCCCTACTGGGAAGTGAGGAGCCCCTCTGCCCGGCCAGCCGCCCCGT
CCGGGAGGGAGGTGGGGGGGTCAGCCCCCCCACCCGGCCAGCCACCCCGTCCGGGAGGGAGGTG
GGGGGGTCAGCCCCCCGCCTGGCCAGCCGCCCCGTCCGGGAGGGAGGTGGGGGGGTCAGCCCTC
CGCCCGGCCAGCCGCCCCGTCTGGGAGGTGAGGGGCGCCTCTGCCCGGCCGCCCCTACTGGGAA
GTGAGGAGCCCCTCTGCCCGGCCAGCCGCCCCGTCCGGGAGGGAGGTGGGGGGGTCAGCCCCCC
GCCCGGCCAGCCGCCCCGTCCAGGAGGGAGGTGGGGGGGGGGGTCAGCCCCCCCGCCCGGACAG
CCGCCCCATCCGGGAGGTGAGGGGCGCCTCTGCCCGGCCGCCCCTACTGGGAAGTGAGGAGCCC
CTCTGCCCGGCCAGCCGCCCCGTCCGGGAGGAGGTGGGGGGGGGGGTCAGCCCCCCGCCCGGCC
AGCCGCCCCGTCCGGGAGGTGAGGGGCGCCTCTGCCCAGCCGCCCCTACTGGGAAGTGAGGAGC
CCCTCTGCCCGGCCAGCCGCCCCGTCCGGGAGGGAGGTGGGGGGGTCAGCCCCCCGCCCGGCCA
GCCGCCCCGTCCGGGAGGTGAGGGGCGCCTCTGCCCGGCCGCCCCTACTGGGAAGTGAGGAGCC
CCTCTGCCCGGCCACCACCCCGTTGGGAGGTGTGCCCAACAGCTCATTGAGAACGGGCCAGGAT
GACAATGGCGGCTTTGTGGAATAGAAAGCGGGAAAGGTGGGGAAAAGATTGAGAAATCGGATGG
TTGCCGTGTCTGTGTAGAAAGAAGTAGACATGGGAGACTTTTCATTTTGTTCTGCACTAAGAAA
AATTCCTCTGCCTTGGGATCCTGTTGATCTGTGACCTTACCCCCAACCCTGTGCTCTCTGAAAC
ATGTGCTGTGTCCACTCAGTGTTAAATGGATTAAGGGCGGTGCAAGATGTGCTTTGTTAAACAG
ATGCTTGAAGGCAGCATGCTCGTTAAGAGTCATCACCAATCCCTAATCTCAAGCAATCAGGGAC
ACAAACACTGCAGAAGGCCGCAGGGTCCTCTGCCTAGGAAAACCAGAGACCTTTGTTCACTTGT
TTATCTGCTGACCTTCCCTCCACTATTGTCCCATGACCCTGCCAAATCCCCCTCTGTGAGAAAC
ACCCAAGAATTATCAATAAAAAAATAAATTAAAAAAAAAAAATTAAAAAAAAAAAAAAAAAAAA
AAAAAAAAAAAAAAAAAAAAAAAAAAAAAAAAGAAAAAAAAAAAAAAAAAAAAAAAAAAAAAAA
AAAAAAAAAAGAAGAAAACCTCTTTTTTATTTTCATTCAACACTTCTGGCCACCAAACAGGTGT
GGGAGTGTTTTCTAAGCAATTCTCCAGTTATCTGGGGACACCAACTGGGTGTCCTACAATTGAA
TCCAATTCTAACACTCTACCTGGAAATAACATCAGATCCCGTGCAGTTTAAGTGCTCCGCCCCA
GCAAATTTCCCCCCGTTTCCAATGTCAGTTACAAGACTGGGTTGTCACCTGTGCATCAGACCAA
CCAGCTATAATTTGGAGATTCCCACAACTCCCTGCTAGGGTTTCATCATTTGCTAGAATAGCTG
ACAAAACTCAGGAGACACTTACATTTACTGGTTTATTATGTAAAGGATTAAGTAAAGGATACAG
ATGAACAGCCAGTTGAAGAGATACATAGGGCAAAATCTGGAAGGGTCCTGGGCCCAGCAGCATC
TATCCTGTGGAGTTCTGTTTGCTACCTTCCTGACACATGTATGTGTTCACCAACCAGAAACTCT
CAGAACCCTGTACTTTAGGAGAAGTCATCATGTAGGCATGATTGATTATTACCCAATCTCCAGC
TCCTCTCTTCTCCCCAGAGGATGGAGAGAGGGGCTGAAAGTTCCAAGCTTCTAATCACACTTGG
TCTTTCTGGTGACCAGCCCCAATCCAGGAGCCAGCAGGAGTAGCCTCATTAGCACAAAAGGCAT
TCCTATGACCCAGGAAATTCCAAGGGATCTAGTAGCTCTGTGTCAGGAACTGGGATCGGAGACC
AAATATTAGATGGTCTAATATTTGGAGCTAAAGATGCTCCTATAGCATATTTTCCCATTATTTC
ACAGCATCATGGCATTGCATATAACCTCATTTTTTCATATATGACCTATCTCTGATCTATTACC
TCACTTCCAAGGTCCTGGGAGATAAGGAGGCAATGACATCATTTTTCAGGGTTGTTTTAGGGAT
GAGGGGCAAGGCCTCTCACCTTGAGAGAGAGTGGCAGTGTTGGGCCCACTAATAGAGGATCCCA
CGAACTGCCAGAGGGTACAGTATGTAATCTGGCAAACAAGGGAAGAGGAAGAGAGCACTGGGAC
TCAGGATGAACCCAGAGAATTCAGGACAAAGGTCTCCATCAGCTGAGGGTATCATGCTGGCTTC
TGACTCTGTTTTTCTCTGGTTTTGCCCT**AG**
